# Whole-Exome Sequencing Identifies Novel Somatic Mutations in Chinese Breast Cancer Patients

**DOI:** 10.4172/1747-0862.1000183

**Published:** 2015-09-25

**Authors:** Yanfeng Zhang, Qiuyin Cai, Xiao-Ou Shu, Yu-Tang Gao, Chun Li, Wei Zheng, Jirong Long

**Affiliations:** 1 Division of Epidemiology, Department of Medicine, Vanderbilt Epidemiology Center, Vanderbilt-Ingram Cancer Center, Vanderbilt University School of Medicine, Nashville, TN, USA; 2 Department of Epidemiology, Shanghai Cancer Institute, Shanghai, China; 3 Department of Biostatistics, Vanderbilt University School of Medicine, Nashville, TN, USA

**Keywords:** Breast cancer, Somatic mutation, Exome sequencing, Chinese population

## Abstract

Most breast cancer genomes harbor complex mutational landscapes. Somatic alterations have been predominantly discovered in breast cancer patients of European ancestry; however, little is known about somatic aberration in patients of other ethnic groups including Asians. In the present study, whole-exome sequencing (WES) was conducted in DNA extracted from tumor and matched adjacent normal tissue samples from eleven early onset breast cancer patients who were included in the Shanghai Breast Cancer Study. We discovered 159 somatic missense and ten nonsense mutations distributed among 167 genes. The most frequent 50 somatic mutations identified by WES were selected for validation using Sequenom MassARRAY system in the eleven breast cancer patients and an additional 433 tumor and 921 normal tissue/blood samples from the Shanghai Breast Cancer Study. Among these 50 mutations selected for validation, 32 were technically validated. Within the validated mutations, somatic mutations in the *TRPM6, HYDIN, ENTHD1*, and *NDUFB10* genes were found in two or more tumor samples in the replication stage. Mutations in the *ADRA1B, CBFB, KIAA2022*, and *RBM25* genes were observed once in the replication stage. To summarize, this study identified some novel somatic mutations for breast cancer. Future studies will need to be conducted to determine the function of these mutations/genes in the breast carcinogenesis.

## Introduction

Breast cancer is the most common malignancy among women worldwide. In the United States, there were 226,870 new cases diagnosed and 39,510 deaths in 2012 [1]. Despite improvements in survival, breast cancer remains the second leading cause of female cancer mortality. It is well-established that breast cancer genomes harbor complex mutational landscapes, often characterized by point mutations, small insertions and deletions (Indels), and large structure changes across the genome [2–7]. Such somatic DNA changes in the tumor genome may result in an inactivation of tumor suppressor genes and activation or deregulation of oncogenes. Comprehensive identification of these somatic changes is necessary to better understand biological mechanisms of carcinogenesis and cancer progression, which may help to predict prognosis and to advance the development of targeted therapies.

To date, large scale genome-wide sequencing projects in breast tumors have identified several key breast cancer related genes, such as *TP53, PIK3CA, PTEN, AKT1, GATA3, CDH1, RB1, MAP3K1*, and *CDKN1B* [3,6]. These studies, however, were conducted in breast cancer patients predominantly of European ancestry. Given the difference in breast cancer subtypes, clinical characteristics, incidence, and survival as well as risk profiles among women of different ethnic backgrounds, somatic aberrations may differ across ethnic groups [8]. For example, compared with lung cancer patients of European ancestry, Asian patients carry more *EGFR* mutations and exhibit higher clinical response rates to *EGFR* tyrosine kinase inhibitors [9]. Similarly, somatic *EGFR* activating mutations in breast cancer were specifically observed in Asian ancestry patients but not in European ancestry patients [10].

To identify novel somatic alternations of breast cancer, we performed whole-exome sequencing in the breast tumor and matched normal DNA samples from eleven Chinese patients. Novel mutations were further selected for replication in additional samples.

## Materials and Methods

### Patients and specimens

The subjects in this study were a subset of participants in the Shanghai Breast Cancer Study (SBCS), a population-based case-control study among Chinese women. Details of the study have been described elsewhere [11,12]. Briefly, cases were women diagnosed with breast cancer between August 1996 and March 1998, 25 to 64 years of age, without a previous cancer diagnosis, and alive at the time of interview. All cases were identified via the population-based Shanghai Cancer Registry. Medical charts were reviewed using a standard protocol to obtain information on cancer treatment, clinical stages, and cancer characteristics, such as estrogen and progesterone receptor status. Two senior pathologists reviewed all tissue slides to confirm the diagnosis. Among the SBCS, most study participants provided blood samples for germline based genetic studies [11,12]. We also collected fresh tumor tissue samples and adjacent non-tumor tissue samples from a portion of the breast cancer patients from the SBCS. During surgery, tumor tissue samples were obtained from the tumor and the adjacent normal tissue samples were obtained from the distal edge of the resection. These samples were snap-frozen in liquid nitrogen as soon as possible, typically within 10 minutes. Samples were stored at −80°C. All patients were interviewed at the time of recruitment.

It has been suggested that patients with early-onset of disease may have a stronger genetic component. In the present study, only patients with early onset of breast cancer were selected for the whole exome sequencing in the discovery stage. The patients were 33 years on average when they were diagnosed with breast cancer. In the replication stage, additional 433 tumor samples and 921 normal tissue/blood samples from the SBCS participants were analyzed. These samples include 158 paired tumor-blood or tumor-normal tissue samples, 275 additional tumor tissue samples, 136 normal tissue samples, and 352 blood samples. Genomic DNA was extracted using a QIAamp DNA kit (Qiagen, Valencia, California) following the manufacturer's protocol. Approval of the study was granted by the relevant institutional review boards in both China and the United States.

### Exome capture, library construction and sequencing

Libraries were constructed by shearing genomic DNA and ligating Illumina paired-end adaptors. The constructed DNA libraries were hybridized to Agilent Human All Exon Target Enrichment kit V1, which was designed to capture 165,637 coding exons and their flanking regions (37.8 million bases, 71.6% in exons with average length of 228 bp). The purified capture products were then amplified and subjected to 72 base paired-end sequencing on the Illumina GAII instrument according to Illumina's standard protocol.

### Read mapping

First, we shifted the Illumina base quality scores (Phred + 64) to the Sanger scale (Phred + 33) [13]. Then, we performed alignment to the human reference genome (hg19) using Burrows-Wheeler Aligner (BWA) algorithm (version 0.7.0) [14] in default parameters. Aligned reads were processed and sorted with SAMtools [15]. We then marked duplicates with Picard (version 1.60) (http://picard.sourceforge.net) and carried out regional realignment and base quality score recalibration (BQSR) using Genome Analysis Toolkit (GATK, version 2.1.8) [16] with default setting. We used QPLOT in default requirement (http://genome.sph.umich.edu/wiki/QPLOT) for sequencing data quality assessment and statistics summary.

### Somatic mutations calling

We conducted two sets of somatic mutations calling. First, we used GATK's Unified Genotyper to call variants simultaneously on all BAM files from the tumor/normal tissue DNA. To decrease the false positive discovery rate, we applied the following criteria to ensure that only high quality reads were used to call and only high quality variants were included in the final analyses: (1) only using reads with a mapping quality score (MAPQ) ≥ 20 and bases with base quality score (BQ) ≥ 20; (2) only including variants with genotype quality score (GQ) ≥ 30 and depth ≥ 30 in both tumor and normal tissue DNA; (3) only including variants exclusively observed in tumor samples.

We also used VarScan2 (v2.3.3) software [17] to identify somatic variants for each paired BAM files from the same patient with default parameters. We also included somatic variants within 100 bp of a target region. In the filter step, we used the somatic Filter command implemented in the VarScan2 program with the following options: -somatic-p-value=0.05 and a minimum of 10× coverage (−min-coverage 10) for both the tumor and matched normal samples. We additionally examined the read pileups for each mutation of each BAM file from both calling sets by using GATK’s Pileup option. Finally, we combined somatic variants called by both GATK toolkit and VarScan2 program for further analyses. We obtained the known somatic mutation dataset (Level 2, version 2.4) in breast cancer studies from TCGA data portal (https://tcga-data.nci.nih.gov/tcga/tcgaHome2.jsp). All known somatic mutations from the COSMIC (v62) were also downloaded. The somatic mutations that were identified in the current study and were not reported in these two datasets were regarded as novel mutations.

### Functional prediction of mutations

All somatic mutations were annotated using SeattleSeq Annotation 137 (http://snp.gs.washington.edu/SeattleSeqAnnotation137/HelpAbout.jsp) and ANNOVAR (http://www.openbioinformatics.org/annovar/). For missense mutations, we combined the SeattleSeq Annotation 137 and SIFT (http://sift.jcvi.org/) to predict the possible impact of an amino acid substitution on the structure/function of the associated protein.

### Mutation verification

To confirm exome sequencing findings, the top 50 somatic mutations ranked by the sequencing depth and frequency were selected for technical validation in the eleven pairs of tumor-normal tissue samples using the Sequenom MassARRAY platform (Sequenom, San Diego, CA). Among these 50 mutations, 32 were validated. They were further investigated in an additional 433 tumor samples and 921 normal tissue/blood samples. PCR primers and allelic-specific extension primers were designed with the MassARRAY Assay Design 4.0 software, and alleles of each mutation were detected through matrix-assisted laser desorption/ionization-time-of-flight mass spectrometry. All calls from Sequenom Typer Analyzer software (version 4.0.20) were manually confirmed by examining the spectra. Peaks for two alleles were checked against the background of each well, and a mutation call was confirmed if the peak was unique to that allele. Blinded duplicates and negative controls (distilled water) were included in each of the 384-well plates.

## Results

### Patient characteristics and sequencing statistics

Clinical characteristics of breast cancer patients included in the present study are listed in Table 1. Patients included in the discovery stage of whole exome sequencing were early-onset with the average diagnostic age of 33 years. Among them, seven were ER+, and three were ER−. Summary statistics for the exome sequencing data are presented in Table S1. For the tumor samples, we obtained an average of 78.28 (range 75.31–82.06) million reads per sample, with a mean depth of 63.39 for the target sequencing regions. On average, 99.34% (99.28–99.46%) of the reads were aligned to the human reference genome. Similarly, for the DNA samples from normal tissue from the same individuals, we got an average of 74.10 (range 61.79–82.27) million reads per sample, with a mean depth of 61.1 across the target regions.

### Somatic mutation profiling

We identified 588 unique somatic point mutations in the study samples (Tables 2 and S2). Each tumor sample carried an average of 63 somatic mutations with a range of 32–133 mutations. The number is comparable to the previous TCGA observation with the mean of 56 mutations [18].

**Figure 1:**
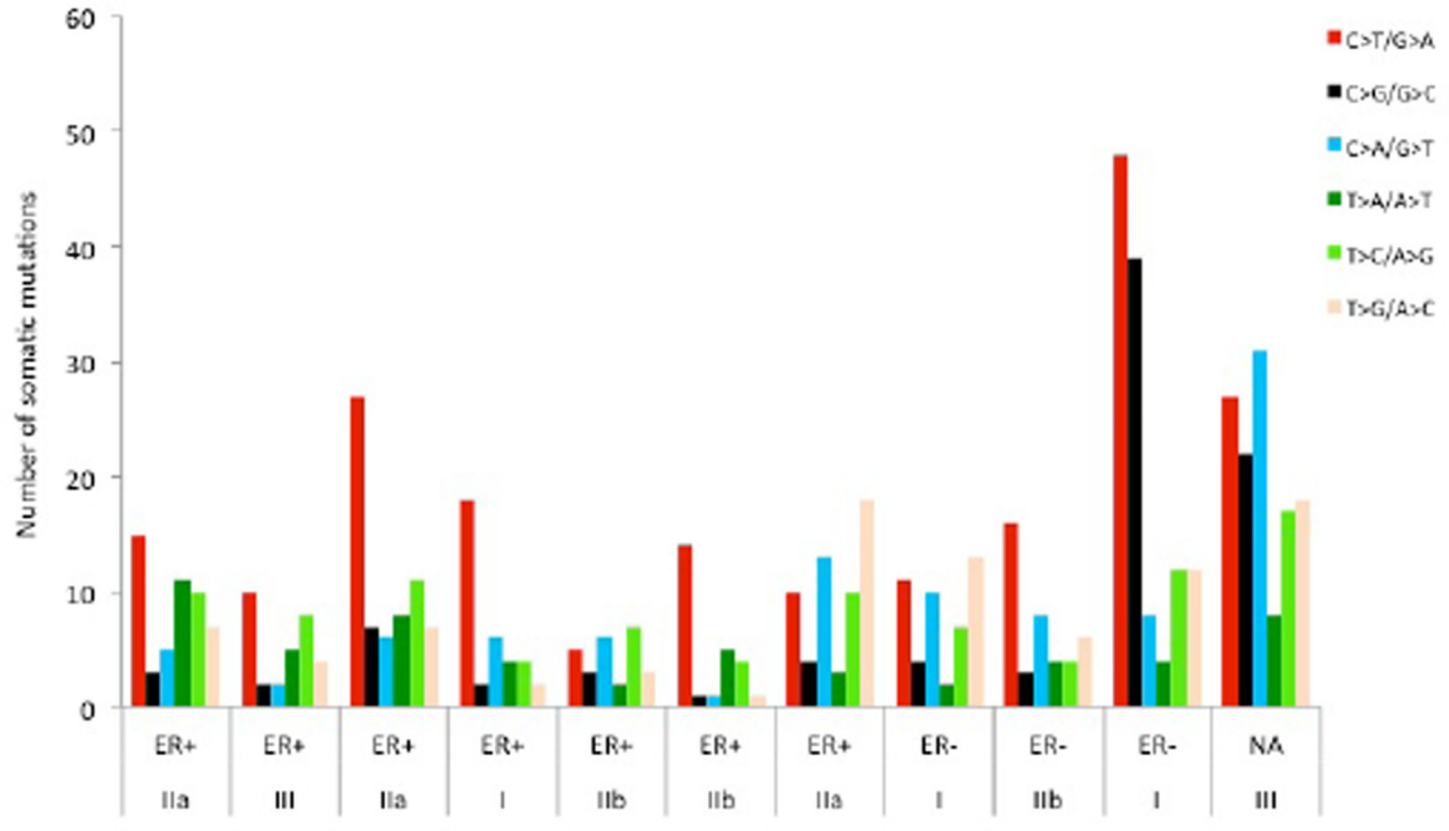
Distribution of somatic mutations with regard to estrogen receptor (ER) status in the discovery stage. The number of mutation types in the target regions is plotted against the ER status and tumor stage. ER+, ER− and NA denote the ER-positive, ER-negative and no available ER status, respectively.

As expected, most mutations (93%) were observed only once. Of the 588 mutations observed, 40.3% were located in coding regions, 4.1% in UTRs, 52.6% in introns, and 3.0% in intergenic regions (Table 2). Among the 237 protein-coding related mutations, 159 were missense, 10 nonsense, 4 splicing, and 64 synonymous mutations (Table 2). Among the six possible mutation classes, including C>T/G>A, C>G/G>C, C>A/G>T, T>C/A>G, T>A/A>T, and T>G/A>C, the C>T/G>A transition dominated the mutation spectra, accounting for 34.2% of the total mutations. The T>A/A>T and C>G/G>C mutations were the least frequent (Figure 1). Such phenomena were observed in ER+ but not in ER− breast cancers. Although only three ER− tumors were surveyed in this study, a similar pattern was reported elsewhere [19]. In addition, we did not find any association between the frequency of somatic mutations and tumor stage (Figure 1). We then focused on the 159 unique missense mutations. They were located in 158 genes, including the known breast cancer genes *PIK3CA, HLA-DPA1, ACACB, FOXD4, KDM3A, CCDC108, ACVR2A, TP53*, and *ATM* (Table S3). The A>G mutation (p.H1047R) and A>T mutation (p.H1047L) in the *PIK3CA* gene were detected in three patients and one patient, respectively, in the discovery stage of whole exome sequencing.

### Validation of novel mutations

We selected 50 missense mutations for validation using the Sequenom MassARRAY system, and 32 of them (64%) were confirmed (Table S4). Furthermore, the *PIK3CA* H1047R mutation was observed in additional 53 patients in the replication stage, including 50 heterozygous and 3 homozygous mutations (Table 3). Of the remaining 31 experimentally validated mutations, three were located in the genes performing mRNA binding and metabolic processes (*KHDRBS1, RBM25, SF3B3*) and four were located in the genes related to phosphate metabolic process (*NDUFB10, TEK, PGK2 and TRPM6*). Besides *PIK3CA* gene, eight of these mutations were observed in independent tumor samples in the replication stage (Table 3).

Of note, the Arg1122Gln mutation in the TRPM6 (transient receptor potential cation channel, subfamily M, member 6) gene was observed in five samples. Both mutations, Ile493Val in the *ENTHD1* (ENTH domain containing 1) gene and Thr3867Ala in the *HYDIN* (axonemal central pair apparatus protein) gene, were observed in an additional three samples. Mutation Glu74Lys in the *NDUFB10* (NADH dehydrogenase (ubiquinone) 1 beta subcomplex, 10, 22kDa) gene was observed in two additional samples, one heterozygote and one homozygote. The other four mutations were observed once in the independent patients in the replication stage, including Ser135Asn in the ADRA1B (adrenoceptor alpha 1B), Leu64Gln in the *CBFB* (core-binding factor, beta subunit), Pro72Ser in the *KIAA2022*, and Lys462Glu in the *RBM25* (RNA binding motif protein 25) (Table 3).

## Discussion

In the present study, we systematically searched somatic alternations in the coding region using samples from eleven Chinese breast cancer patients. We identified 588 mutations with 40% located in coding regions. Of the 50 mutations selected for validation, 32 were technically validated. Nine mutations were detected in independent samples in the replication stage. Especially, mutations in the *PIK3CA, TRPM6, HYDIN, ENTHD1* and *NDUFB10* genes were observed in multiple samples in the replication stage.

One of the most significant findings was the recurrent R1122Q mutation in the *TRPM6* gene, which was detected in 6 of 444 breast cancer tumor samples in the present study. The *TRPM6* gene plays a crucial role in magnesium homeostasis and epithelial magnesium transport. Both basic and pre-clinical studies show that the magnesium flux by TRP magnesium channels is highly related with tumor cell proliferation and cycle, angiogenesis, tumor growth and reprogramming, as well as metastasis [20]. The R1122Q mutation occurs at the topological domain of *TRPM6* towards the cytoplasm, according to the Uniprot database annotation (http://www.uniprot.org/uniprot/Q9BX84). Thereby, dysfunction of the *TRPM6* protein may affect the cation homeostasis leading to the tumor development. Recently, Stephens et al. reported two somatic mutations (p.T1822A and p.A1765T) in the *TRPM6* gene from screening 100 breast cancer patients of European ancestry [6]. However, the mutation R1122Q that was observed in the present study was not reported in the study of Stephens et al. [6]. Meanwhile, another study, which included 108 breast cancer patients from Mexico and Vietnam using whole-genome and whole-exome sequencing, did not discover any mutations in the *TRPM6* gene [21]. Mutations in other members of the *TRPM* gene family were also reported in breast cancer tumor genome, including three mutations in each of the *TRPM8, TRPM3*, and *TRPM1* genes [6]. In addition, mutations in the *TRPM6* gene were also discovered in patients of non-tumor diseases, such as hypomagnesemia with secondary hypocalcemia [22].

In addition to the observed recurrent missense mutation (T3867A) in the *HYDIN* gene in the present study, ten other mutations have been reported in previous studies [6,21], though each was observed only once in 100 breast cancer patients. The mutation site (T3867A) observed in the present study shows evolutionarily high conservation across all vertebrates with 4.26 of Genomic Evolutionary Rate Profiling (GERP) score, implying potentially functional constraint of this mutation site in the *HYDIN* gene. However, there is less direct evidence linking the biological role of the *HYDIN* gene to breast carcinogenesis. It will be of interest to determine the mechanistic and phenotypic consequences of *HYDIN* mutations in the breast and other tumor cells. We also detected a recurrent mutation (I493V) in the *ENTHD1* gene of four patients. However, in the previously two exome sequencing studies of breast cancer, no *ENTHD1* mutations were observed [6]. The functional feature of this gene is largely unknown. A recent study showed that the *ENTHD1* protein might interact with *HDAC6* [23], suggesting that the *ENTHD1* may be associated with the histone acetyltransferase activity.

In the present study, we also found recurrent novel missense mutations in the *CBFB, ADRA1B, KHDRBS1, NDUFB10, RBM25*, and *KIAA2022* genes. Among them, the *CBFB* oncogene has been documented into the COSMIC cancer Gene Census, a gene category for which mutations have been causally implicated in cancer [24]. The *CBFB* gene plays a critical role in tumor cell growth and proliferation [25]. Other mutations in this gene were recently reported in breast cancer patients, including nonsense mutations at E152 and R83 and truncating frame shift mutations at E16, Q67 and Y96 [6, 21]. The *CBFB* mutations were found to be accompanied by deletions of the gene encoding its binding partner, *RUNX1*, in some breast cancers [21]. For the *ADRA1B* gene, which encodes adrenoceptor alpha 1B, a previous study showed this gene is highly expressed in breast tumors with increased tumor recurrence and poor clinical outcome [26], suggesting this gene may perform a cancer-promoting role. The *KHDRBS1* gene encodes KH domain containing, RNA binding, signal transduction associated 1. The protein appears to have many functions and may be involved in a variety of cellular processes, including alternative splicing, cell cycle regulation, RNA 3'-end formation, and tumorigenesis [27]. The *NDUFB10* gene encodes NADH dehydrogenase (ubiquinone) 1 beta subcomplex 10, an integral factor in Complex 1 on the mitochondrial inner membrane [28]. Phosphorylation of NDUFB10 by Src kinase could participate in the development of the proliferative phenotype of glycolytic cancer cells, such as 143B and DU145 cells by preserving complex I activity [29]. However, no mutations were observed in the previous breast cancer exome sequencing data [6]. The *RBM25* gene encodes the RNA binding motif protein 25, one of the RNA-binding regulators that direct the alternative splicing of apoptotic factors such as Bcl-x [30]. Three other mutations, R708Q, H6D, and G130A, were observed in one study [21] but not in another study [6]. A different mutation, *R348Q*, in the *KIAA2022* gene is observed in breast cancer tumor in a previous study [21]. Taken together, the discovery of some novel somatic mutations partially indicate the difference of somatic aberrations across ethnic groups of patients. Further comprehensive comparison in genomic aberrations may help to develop cancer therapeutics strategies for the ethnic-specific groups of patients.

There are several limitations in this study. The small sample size in the discovery stage of the present study limits the power to detect driver mutations and compare the mutation profiles stratified by disease subtype. Biological samples used in the present study were collected a couple of years ago, which may affect somatic mutations discovery. However, in the present study, we observed those recurrent somatic mutations such as the H1047R mutation in the *PIK3CA* gene. Another limitation is that only a small portion of mutations were selected for replication. In addition, there are some differences in patient characteristics between the discovery and replication stage, such as age and ER/PR status. Small Indels as well as large structure changes were not investigated in the present study due to the high false positive rate using exome sequencing [31]. No data were available for tumor purity, which may affect the mutation discovery and validation. Similar to other exome sequencing projects, although the high density of read coverage across target region is obtained from the whole-exome sequencing, with over 60-fold on average, there is a small portion of the coding regions in low or no read coverage due to the capturing design.

In summary, we discovered novel somatic mutations in the *TRPM6, HYDIN, ENTHD1* and *NDUFB10* genes in early onset Chinese breast cancer patients through whole exome sequencing. These mutations were replicated in independent breast cancer patients. Our findings of these novel mutations may provide new clues regarding the molecular mechanism of breast cancer carcinogenesis.

## Figures and Tables

**Table 1:** Clinical characteristics of breast cancer patients in the discovery and replication stages.

Characteristics	Discovery stage	Replication stage
No. of breast cancer patients with tumor tissue samples	11	433
Age of diagnosis (median, range)	33.1 (28–42)	49.4 (26.1–74.2)
**TNM (%)**		
0–I	27.27	30.02
II	54.55	55.89
III	18.18	7.62
IV	0.00	0.46
Unknown	0.00	6.00
**ER status (%)**		
Positive	63.64	61.20
Negative	27.27	27.25
Unknown	9.09	11.55
**PR status (%)**		
Positive	72.73	56.58
Negative	18.18	31.18
Unknown	9.09	12.24
**Chemotherapy (%)**		
Yes	90.91	91.22
No	9.09	5.08
Unknown	0.00	3.70
**Radiotherapy (%)**		
Yes	36.36	36.26
No	36.36	51.96
Unknown	27.27	11.78
**Hormonal therapy (%)**		
Yes	54.55	57.97
No	18.18	32.33
Unknown	27.27	9.70

**Table 2:** Number of somatic mutations identified in the discovery stage using whole exome sequencing.

Mutation class	Breast cancer patients											Sum	Total unique	Percent age (%)
	1	2	3	4	5	6	7	8	9	10	11			
Missense	12	5	22	10	3	5	6	35	14	49	13	174	159	27
Splice site	0	0	0	1	0	0	0	1	0	2	0	4	4	0.7
Nonsense	0	0	0	4	0	0	1	2	1	2	0	10	10	1.7
Synonymous	4	2	7	2	4	3	3	15	8	17	5	70	64	10.9
Intron	30	21	29	16	32	18	13	63	29	50	27	328	309	52.6
3'/5' UTR	1	1	4	3	2	0	2	5	1	3	2	24	24	4.1
Intergenic	4	2	4	0	0	0	1	2	5	0	0	18	18	3

**Table 3:** Replication of the somatic mutations in independent breast cancer patients.

Gene	Chromosome	Position	Mutation	Amino acid change	No. §
ADRA1B	5	159344316	G>A	p.Ser135Asn	1
CBFB	16	67070567	T>A	p.Leu64Gln	1
ENTHD1	22	40140031	T>C	p.Ile493Val	3
HYDIN	16	70896126	C>T	p.Thr3867Ala	3
KIAA2022	X	73964178	G>A	p.Pro72Ser	1
NDUFB10	16	2011243	G>A	p.Glu74Lys	1 (1)*
PIK3CA	3	178952085	A>G	p.His1047Arg	50 (3)*
RBM25	14	73572910	A>G	p.Lys462Glu	1
TRPM6	9	77390837	C>T	p.Arg1122Gln	5

§ No. of patients carrying the mutation

* The number of homozygote detected.

## Abbreviations

BQ: Base Quality Score
BQSR: Base Quality Score Recalibration
BP: Base pairs
GQ: Genotype Quality Score
Indels: Inserts and deletions
MAPQ: Mapping Quality Score
SBCS: Shanghai Breast Cancer Study
